# Relapsing Neurological Complications in a Child With ATP1A3 Gene Mutation and Influenza Infection: A Case Report

**DOI:** 10.3389/fneur.2021.774054

**Published:** 2021-12-15

**Authors:** Raffaella Pisapia, Nicolina Capoluongo, Giulia Palmiero, Carlo Tascini, Carolina Rescigno

**Affiliations:** ^1^UOC Neurological Infectious Diseases, AO dei Colli, Cotugno Hospital, Naples, Italy; ^2^Infectious Diseases Clinic, Udine University Hospital, Udine, Italy

**Keywords:** ATP1A3 gene mutations, influenza, encephalopathy, differential diagnosis, case report

## Abstract

Mutations in the ATP1A3 gene encoding the α3 subunit of Na+/K+-ATPase are associated with different neurological manifestations that may be elicited by febrile episodes. A recently described phenotype, linked to the p.Arg756Cys mutation, is clinically characterized by Relapsing Encephalopathy with Cerebellar Ataxia (RECA). In our case, a diagnosis of RECA has been established, and despite an alternative, reasonable cause had been already identified.

We describe the case of a child with two recurrent episodes, 2 years apart, of hypotonia and ataxia. In both episodes, a laboratory-confirmed influenza virus infection suggested the diagnosis of influenza-associated encephalopathy. After the second episode, a search for genetic mutations was performed, and ATP1A3 mutation associated to RECA was found. After both episodes, the child was discharged after partial improvement of neurological conditions.

The diagnosis of encephalopathy in children is often challenging. A genetic predisposition to neurological decompensation should be suspected in case of recurrent episodes, even if an alternative diagnosis has been established. Indeed, febrile infections may only represent the trigger of neurological involvement. In these patients, the knowledge of a genetic predisposing factors may help in the prevention of neurological episodes by the prompt use of anti-pyrectics and preventive measures as appropriate vaccination.

## Introduction

In recent years, the role of mutations in the ATP1A3 gene, encoding the α3 subunit of Na^+^/K^+^-ATPase, have been discussed and increasingly described in literature (1, 2). Three main syndromes have been associated to these mutations: Alternating Hemiplegia of Childhood (AHC), Rapid-onset Dystonia Parkinsonism (RDP), and CAPOS syndrome (cerebellar ataxia, areflexia, pes cavus, optic atrophy, and sensorineural hearing loss). From 2004 to 2012 both autosomal dominant and *de novo* mutations in ATP1A3 have been detected in patients affected by these three conditions (3–6).

Other clinical presentations of ATP1A3 mutations that do not fall within one of these major syndromes have also been reported and new phenotypes have been described (7). A new phenotype linked to the p.Arg756Cys mutation, characterized by relapsing encephalopathy with cerebellar ataxia, named RECA has been recently identified (8). Clinical manifestations of these mutations may be elicited by infective triggers or febrile episodes.

Also, influenza virus is a cause of encephalitis or encephalopathy. Although rare, neurological involvement may occur especially in children and is characterized by a broad spectrum of manifestations, including movement disorders and ataxia (9, 10). These manifestations are similar to those observed among children with ATP1A3 mutations.

We describe a case report of a child who experienced two recurrent episodes of encephalopathy, 1 year apart, apparently associated to influenza infection. After the second episode, despite the positivity of nasal swab for influenza, a genetic analysis was performed, and a diagnosis of ATP1A3 mutation was made.

## Case Description

An 18-month-old male child, with no underlying medical conditions, presented in January 2018 with a history of 4 days of fever reaching 39°C and cough, followed on day 2 by irritability and decreased muscle tone.

The child was fully vaccinated according to the Italian vaccination schedule (11), and met all developmental milestones until the onset of the symptoms. The child was not vaccinated for seasonal influenza. His parents and his older sister were healthy.

On admission, he was whiny and showed hypotonia of the four limbs with poor head control and inability to maintain a sitting position. No signs of meningeal irritation were present and cranial nerve examination was normal. A few days later, the child developed ataxic gait.

Blood testing revealed white blood count 4,600/mm3 with neutrophil at 42.6%, lymphocytes at 42.6%, monocytes at 14.8%, hemoglobin: 12.1 g/dl, platelet counts: 257.000/mm3, and C-reactive protein:0.2 (normal value 0–0.3) mg/dl. Biochemical investigations, including serum liver and kidney function tests and electrolytes were normal. Multiple Polymerase chain reaction (PCR) (Seeplex®RV15 one step and Seeplex® Pneumobacter Ace detection) performed on nasal swab for common bacterial and viral respiratory infections resulted positive for Influenza A-H1N1 pdm09, *Streptococcus pneumoniae*, and *Haemophilus influenzae*.

A chest radiograph evidenced a parenchymal consolidation, and computed tomography (CT) scan of the brain revealed no pathologic lesions.

Cerebrospinal fluid (CSF) was clear and colorless, and its examination showed 4 cells, protein of 29 mg/dl, and glucose of 65 mg/dl (glycemia 105 mg/dl). CSF cultures were bacteriologically sterile. Multiple PCR (Biofire® Film Array Meningitis Encephalitis (FAME) was negative for the most frequent viruses, bacteria causing meningo-encephalitis, and further microbiologic workup of CSF for other viruses and bacteria (influenza, parainfluenza 1–2–3–4, adenovirus, metapneumovirus, Epstein Barr virus, mycoplasma pneumoniae, Chlamydophila pneumoniae) and acid-fast bacilli bacteria were negative.

The electroencephalogram (EEG) showed a picture of severe cortico-subcortical suffering.

Contrast enhanced magnetic resonance imaging (MRI) demonstrated slight vermian cistern enlargement. The patient was diagnosed with influenza-associated encephalopathy (IAE) based on the clinical findings. He was treated with oral oseltamivir, intravenous ceftriaxone, pulse dexamethasone, and levetiracetam.

After 18 days of hospitalization, the child was discharged in improved clinical conditions, i.e., resolution of trunk and head hypotonia, while persisting cerebellar ataxia without impairment of cognitive functioning.

In January 2020, the same child returned to our observation because of influenza-like symptoms (fever and cough) associated with difficulty in maintaining upright position and ataxic gait.

On admission, physical examination showed absence of meningeal signs, irritability, mild positive red dermographism, and absence of osteotendinous reflexes.

Blood exams, including blood count, C- reactive protein, liver, and kidney functions, were all in the normal range.

Multiple PCR (Seeplex®RV15 one step and Seeplex® Pneumobacter Ace detection) performed on nasal swab resulted positive for influenza AH3N2.

Cerebrospinal Fluid (CSF) analysis showed: 2 cells, protein 25 mg/dl and glucose 70 mg/dl (glycemia 180 mg/dl). Multiple PCR (Biofire® Film Array Meningitis Encephalitis (FAME) was negative along with PCR for influenza virus and the search for other infectious causes of encephalitis (parainfluenzae viruses, adenovirus, parvovirus B19, Epstein Barr virus, acid fast bacilli bacteria). CSF culture was also negative.

The search of autoimmune and paraneoplastic markers of encephalitis (anti-NMDA, anti-Hu, anti-Yo, Anti-Ri, Anti-CV2, Anti-Ma2, anti-amphiphisin) were investigated and were all negative. Brain MRI was normal, and the EEG showed sleep-related brain physiological activity.

Treatment with oseltamivir and pulse dexamethasone was started. Furthermore, in the hypothesis of an immune mediated mechanism as a pathogenetic component of IAE, intravenous immunoglobulin was added. Clinical conditions progressively improved with reduction of tremors and trunk oscillations and more coordinated walking.

However, despite improvement, this second episode raised suspicion of a genetic predisposition to neurological manifestation triggered by specific events, so a single nucleotide polymorphism (SNP) array was performed, revealing the heterozygous *de novo* mutation c.2266C > T (p.Arg756Cys) of ATP1A3 gene.

The child was discharged on day 16 with slight ataxic gait.

## Discussion

Influenza is a common diagnosis during the winter season. Despite how it is mostly a self-limiting mild disease, primarily affecting upper respiratory tract, neurological complications may occur, especially among children. These complications include encephalopathy/encephalitis, seizures, transverse myelitis, acute disseminated encephalomyelitis, and Guillain-Barré syndrome. Cerebellar involvement has also been described with generalized hypotonia and ataxia, as occurred in our patient (1, 2). For this reason, a diagnosis of Influenza associated encephalopathy (IAE) has been made after the first episode, with no further research for alternative or concomitant causes.

A diagnosis of IAE is also possible in the absence of Influenza virus isolation in cerebrospinal fluid (CSF). Indeed, in agreement with literature data, influenza virus is rarely neurotropic and rarely found in CSF. The pathogenesis of these manifestation seems to be driven by immunologic and/or metabolic processes damaging vascular endothelium and causing inflammation and apoptosis of vascular endothelium and brain tissue (“cytokine storm”) (12). For these reasons, the term encephalopathy is often preferred (13, 14).

The clinical characteristics of the recurrent episode, despite the hypothesis of influenza encephalopathy was made again, stimulated us to explore alternative diagnosis or the presence of predisposing factors of neurological decompensation, including genetic mutations.

Genetic analysis revealed the presence of the heterozygous ATP1A3 gene mutation, specifically the p.Arg756Cys variant.

Mutations in ATP1A3 gene lead to different phenotypes having in common acute neurological decompensation episodes triggered by different factors, including febrile episodes. In our case, the Influenza could only have acted as a trigger, probably as a febrile illness, and not itself the cause of the encephalopathy.

Alongside the well-characterized clinical phenotypes AHC, RDP, and CAPOS, a new phenotype clinically characterized by relapsing encephalopathy with cerebellar ataxia (RECA) has been firstly diagnosed in an adult in 2015 and associated with p.Arg756Cys variant (15). Since then, several cases have been described in children.

The main characteristic is hypotonia associated to areflexia and ataxia. However, the severity of symptoms can be variable along with the long-term sequelae. In fact, each episode is followed by slow and partial recovery. Also, for our case, persisting weakness, tremor in the limbs, and ataxia were present at discharge and required, after both episodes, motor rehabilitation.

Our description aims to enrich the literature of clinical cases related to ATP1A3 gene mutations in consideration of recent discovery of this phenotype. Furthermore, it underlines that even if a primary diagnosis has been established, in the presence of recurrent episodes, a genetic evaluation is always advisable and should be considered. Indeed, the knowledge of a genetic predisposing factor may help in the prevention of neurological episodes, e.g., by the prompt use of anti-pyretic drugs. Specifically, in our case, the seasonal influenza vaccination would represent an effective protective measure.

## Data Availability Statement

The original contributions presented in the study are included in the article/Supplementary Material, further inquiries can be directed to the corresponding authors.

## Author Contributions

RP conceived and drafted the paper and performed literature search. NC and GP performed literature search and contributed for important intellectual contents. CT and CR revised the paper and contributed for important intellectual contents. All authors participated in clinical and diagnostic management of the patient, and approved the final version of the paper.

## Conflict of Interest

The authors declare that the research was conducted in the absence of any commercial or financial relationships that could be construed as a potential conflict of interest.

## Publisher's Note

All claims expressed in this article are solely those of the authors and do not necessarily represent those of their affiliated organizations, or those of the publisher, the editors and the reviewers. Any product that may be evaluated in this article, or claim that may be made by its manufacturer, is not guaranteed or endorsed by the publisher.
