# Supplementary material for: Relapsing Neurological Complications in a Child With ATP1A3 Gene Mutation and Influenza Infection: A Case Report
Source: Front Neurol. 2021 Dec 15;12:774054. doi: 10.3389/fneur.2021.774054 (PMC8715801; doi:10.3389/fneur.2021.774054)
Supplement: Supplementary file 1 [file Data_Sheet_1.PDF]

CARE case report guidelines

CARE Checklist of information to include when writing a case report

(cc) BY-NC-ND

Topic	Item	Checklist item description	Reported on Line
Title	1	The diagnosis or intervention of primary focus followed by the words “case report”	1-2
Key Words	2	2 to 5 key words that identify diagnoses or interventions in this case report, including "case report"	13-14
Abstract (no references)	3a	Introduction: What is unique about this case and what does it add to the scientific literature?	20-21
	3b	Main symptoms and/or important clinical findings	22
	3c	The main diagnoses, therapeutic interventions, and outcomes	22-26
	3d	Conclusion—What is the main “take-away” lesson(s) from this case?	30-32
Introduction	4	One or two paragraphs summarizing why this case is unique (**may include** references)	50-53
Patient Information	5a	De-identified patient specific information	56
	5b	Primary concerns and symptoms of the patient	57-58; 88-91
	5c	Medical, family, and psycho-social history including relevant genetic information	59-61
	5d	Relevant past interventions with outcomes	56
Clinical Findings	6	Describe significant physical examination (PE) and important clinical findings	62-64: 91-92
Timeline	7	Historical and current information from this episode of care organized as a timeline	NA
Diagnostic Assessment	8a	Diagnostic testing (such as PE, laboratory testing, imaging, surveys).	65-83; 93-104
	8b	Diagnostic challenges (such as access to testing, financial, or cultural)	104-112
	8c	Diagnosis (including other diagnoses considered)	83-84; 109-112
	8d	Prognosis (such as staging in oncology) where applicable	NA
Therapeutic Intervention	9a	Types of therapeutic intervention (such as pharmacologic, surgical, preventive, self-care)	84-85; 105-107
	9b	Administration of therapeutic intervention (such as dosage, strength, duration)	NA
	9c	Changes in therapeutic intervention (with rationale)	NA
Follow-up and Outcomes	10a	Clinician and patient-assessed outcomes (if available)	86-88; 113
	10b	Important follow-up diagnostic and other test results	109-112
	10c	Intervention adherence and tolerability (How was this assessed?)	NA
	10d	Adverse and unanticipated events	NA
Discussion	11a	A scientific discussion of the strengths AND limitations associated with this case report	146-152
	11b	Discussion of the relevant medical literature **with references**	134-141
	11c	The scientific rationale for any conclusions (including assessment of possible causes)	116-133
	11d	The primary “take-away” lessons of this case report (without references) in a one paragraph conclusion	147-152
Patient Perspective	12	The patient should share their perspective in one to two paragraphs on the treatment(s) they received	NA
Informed Consent	13	Did the patient give informed consent? Please provide if requested	Yes ☒ No ☐
